# Darbepoetin Versus Epoetin Alfa for the Correction of Anemia in Cancer Patients Receiving Radiotherapy or Chemoradiotherapy Treatment

**DOI:** 10.4137/cmo.s510

**Published:** 2008-05-19

**Authors:** Pilar Ma Samper Ots, Concepción López Carrizosa, Aurora Rodríguez Pérez, Juan de Dios Saez Garrido, José Ma Delgado Pérez

**Affiliations:** Department of Oncological Radiotherapy Hospital Central de la Defensa, Madrid, Spain

**Keywords:** darbepoetin alfa, epoetin alfa, anemia, radiotherapy, radiochemotherapy

## Abstract

**Introduction:**

Anemia is the most frequent hematological disturbance in cancer patients, with prevalence between 30% and 90%, depending on the type of tumor, the antitumor treatment, and other factors (infection, malnutrition, bleeding, tumor infiltration of the bone marrow). A number of erythropoietic agents have shown to be effective in increasing the hemoglobin (Hb) levels, reducing the requirements for transfusion, and improving quality of life. The objective of this study is to compare darbepoetin alfa and epoetin alfa when used to correct anemia in cancer patients who are receiving radiotherapy or radiochemotherapy.

**Material and methods:**

A prospective study of 125 consecutive patients with anemia (Hb <13 g/dL in males or <12 g/dL in females) who were undergoing treatment with radiotherapy (RT) or radiochemotherapy (RCT) in our department were enrolled between March 2003 and March 2005. The treatment for the anemia was either darbepoetin alfa 150 mcg/week (62 patients, group 1) or epoetin alfa 40,000 IU/week (63 patients, group 2). Patients received iron supplements in both groups. Treatment was administered in a consecutive manner depending on tumor type. If the increase in Hb was <1 g/dL after 4 weeks of treatment, the dose was increased to 300 mcg/week in group 1 or to 60,000 IU/week in group 2. The treatment was terminated when a Hb value of ≥15 g/dL was reached during RT treatment, a Hb value of ≥14 g/dL was reached if the RT had been completed, or after 16 weeks of treatment whatever the Hb value. The mean age of patients was 63.36 ± 11.27 years, 67% were male. No significant differences were observed between the 2 groups in tumor type or stage, previous treatments, or intent to treat with RT or RCT.

**Results:**

Comparing group 1 and group 2 by intent to treat, the mean Hb at the start of treatment with the study drug was 12.1 g/dL vs 11.8 g/dL, the proportion of patients whose dose was increased was 19.7% vs 24.6%, the need for transfusion was 3.2% in each group, the duration of erythropoietic treatment was 6.5 weeks in both groups, and 2 patients in group 2 restarted treatment with epoetin alfa. The percentage of patients who responded (defined as an increase in the Hb ≥ 2 g/dL in the absence of transfusions) was of 72.6% and 66.7%, respectively. Four vascular adverse events were observed, 2 in each group. No significant differences were observed with respect to the baseline, week 4, and week 12 levels of endogenous erythropoietin, serum iron,% saturation, and ferritin. The increase in Hb 1 month after the final administration of the study drug was 2.21 g/dL in group 1 and 2.46 g/dL in group 2 (p = ns).

**Conclusions:**

The results of our study demonstrate that both treatments are equally effective in correcting anemia in cancer patients undergoing RT or RCT.

## Introduction

Anemia is present in 30% to 90% of cancer patients, depending on the type of tumor and on the definition of anemia used (1). Its origin is multifactorial: chronic inflammation, blood loss, nutritional deficits, hemolysis, bone marrow infiltration by malignant cells, low erythropoietin levels, and a reduction in the response to erythropoietin (2). Furthermore, most oncological treatments cause anemia (3). Anemia increases tumor hypoxia, which is common in patients with anemia, may increase the aggressive behavior of the tumor and reduce the efficacy of radiotherapy (4–6), and is therefore a prognostic factor for local control and survival (7–11).

The current treatment of anemia in cancer patients is the administration of erythropoiesis-stimulating factors. In cases of severe anemia, red blood cell (RBC) transfusions may also be used. Transfusions provide a rapid correction of anemia but are associated with a series of risks such as acute transfusion reactions and possible infections (12).

Recombinant human erythropoietin (rHuEPO) is a protein that stimulates erythropoiesis and is effective in the prevention and reversal of anemia in patients with cancer (13). Darbepoetin alfa also stimulates erythropoiesis and has a longer serum half-life than rHuEPO. Darbepoetin alfa can be used in various doses and regimens including administration every 1, 2, or 3 weeks. It is well tolerated and effective for the treatment of anemia in cancer patients (14,15).

The objective of this study was to compare darbepoetin alfa with epoetin alfa, both administered weekly, for the correction of anemia in cancer patients undergoing radiotherapy (RT) or radiochemotherapy (RCT) treatment.

## Material and Methods

We performed a prospective study, between March 2003 and March 2004, that included 125 consecutive patients with anemia, defined as a Hb <13 g/dL in males or <12 g/dL in females. These patients were undergoing either RT or RCT in our department. Basal endogenous erythropoietin (EPO) levels and iron metabolism were measured at baseline and during the 4th and 12th weeks; and a weekly blood count was measured during RT or RCT treatment and one month after the final dose of the study drug. Two treatment groups were established: group 1, darbepoetin alfa 150 mcg/week; and group 2, epoetin alfa 40.000 IU/week. Oral iron supplements were given to patients in both groups. If the Hb increase was <1 g/dL after 4 weeks of treatment, the dose was increased to 300 mcg/week in group 1 or to 60.000 IU/week in group 2. The treatment was suspended if Hb values ≥15 g/dL were reached during RT or RCT treatment, if values ≥14 g/dL were reached after completing the RT or RCT, or after 16 weeks of treatment regardless of the Hb level. RBC transfusion was indicated if the Hb value fell below 8 g/dL. Assignment to treatment groups was made in a consecutive manner according to the tumor type: 62 patients were assigned to group 1 and 63 patients to group 2. The mean age overall was 63.36 ± 11.27 years, and 67% were males. The descriptive characteristics of the sample are presented in Table 1. Both groups well are balanced and not statistically significant differences were observed between the groups with respect to age, tumor type or stage, previous oncological treatment, or current oncological treatment: RT or RCT and intent to treat with RT.

The following variables were analyzed:

- the changes in Hb levels,
- the proportion of patients who doses were increased,
- the percentage of patients who responded (defined as an increase in Hb of ≥2 g/dL in the absence of RBC transfusions),
- the duration of treatment with the study drug,
- the transfusion requirements,
- the changes in iron metabolism, and
- the onset of adverse events.

The SPSS version 12.0 program was used for the statistical analysis. The results are presented by intent to treat. The descriptive statistic of the sample has been made as well as comparison of the groups by means of the ANOVA test, X2 de Pearson, and univariante analysis to determine the factors that influence in the response to the treatment. The p meaning level has been considered ≤ to 0.05.

## Results

**Figure 1:**
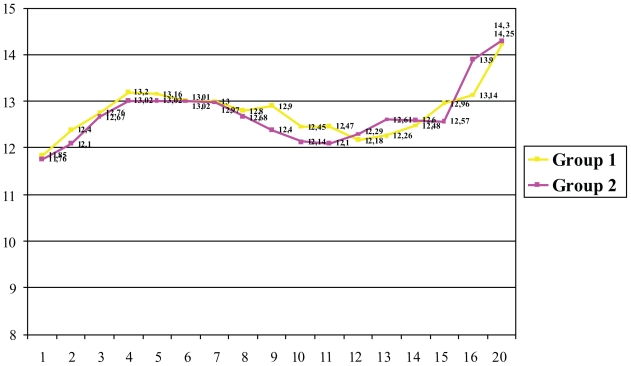
Changes in the Hb over the course of the study.

The mean levels of endogenous EPO were of 45.03 ± 44.02 mU/mL (95% confidence interval of the mean: 37.04 to 53.02 mU/mL). No statistically significant differences were observed between treatment groups (p = 0.076). The mean Hb level at the start of treatment with the study drug was 11.85 ± 1.12 g/dL in group 1 and 11.76 ± 1.13 g/dL in group 2 (p = 0.651). After 4 weeks of treatment, the Hb levels had increased in both groups: 13.16 ± 1.7 g/dL in group 1 and 13.02 ± 1.93 g/dL in group 2 (p = 0.320). The weekly changes in the Hb levels are shown in Figure 1. In group 1, the darbepoetin alfa dose was doubled in 12 patients (19.4%). The epoetin alfa dose was doubled in 15 patients (23.8%) in group 2 (p = 0.667). During the study, 2 patients (3.2%) from group 2 restarted treatment with epoetin alfa. The percentage of patients who responded was 72.6% (45 patients) in group 1 and 66.7% (42 patients) in group 2 (p = 0.3).

The mean treatment duration with the study drug was 6.47 ± 4.09 weeks in group 1 and 6.5 ± 4.1 weeks in group 2 (p = 0.923). The erythropoietic agent total average dose administered in each group was of 970.5 mcg for darbopoetin alpha and 260,000 UI for epoetin alpha respectively. The reason for termination of treatment with the study drug are presented in Table 2.

One month after the final dose of the epoetin alfa or darbepoetin alfa, the mean Hb levels were 14.25 ± 1.08 g/dL, an increase of 2.21 g/dL over baseline, in group 1 and 14.3 ± 1.35 g/dL, an increase of 2.46 g/dL over baseline, in group 2 (p = 0.440).

The Hb levels at the start of RT or RCT treatment was 13.01 ± 1.43 g/dL in group 1 and 12.7 ± 1.77 g/dL in group 2 (p = 0.461) and at the end of RT or RCT were 13.47 ± 1.69 g/dL and 13.2 ± 1.89 g/dL, respectively (p = 0.415).

Thirty-four adverse events (27,4% in group 1 and 27% in group 2, p = 0.558) were observed. Most adverse events were related to the tumor progression or the oncological treatment. However, 4 adverse events (2 in each treatment group) were vascular and may have been related to the administration of the study drug: 1 thrombophlebitis event, 2 acute myocardial infarction events, and 1 aortic aneurysm rupture. All events except thrombophlebitis resulted in death. Blood pressure was controlled weekly during the study and showed no marked changes.

The mean levels of serum iron, ferritin, and % saturation are shown in Table 3.

## Discussion

Recombinant HuEPO is useful in the treatment of anemia, increasing the hemoglobin levels and reducing the need for transfusion in patients with chronic anemia due to cancer. It also improves the quality of life and the performance status and is well tolerated (16,17). The best results have been obtained by subcutaneous administration (18). The standard dose of epoetin alfa in cancer patients is 150 IU/kg, 3 times per week, doubling the dose if the Hb levels have not increased at least 1 g/dL after 4 weeks of treatment. However, pharmacokinetic and pharmacodynamic studies have demonstrated equivalence between a weekly regimen of 40,000 IU and a thrice weekly regimen of 150 IU (19). In 2001, Gabrilove et al. (20), published the results from 3012 non-hematologic cancer patients undergoing chemotherapy treatment who were also receiving epoetin alfa at doses of 40,000 IU/week. The dose was increased to 60,000 IU/week if no response was obtained after 4 weeks. This once weekly regimen increased the Hb levels, reduced the transfusion requirements, and improved the quality of life in patients with cancer and anemia who were receiving chemotherapy. The results were similar to those seen when a three times a week regimen was used.

Darbepoetin alfa, with its higher concentration of sialic acid, has a longer mean half-life than other epoetins. Several randomized studies have demonstrated its efficacy and tolerance in the control of anemia induced by chemotherapy in patients with lymphoproliferative diseases (21) and in those with solid tumors (15) when given weekly or three times a week (22).

In our study, we have used the guideline of administration of weekly erythropoietic agent, since the obtained results are equal to the obtained ones with regimes of three days per week and the quality of life of the patient is improved. Oral iron was administered to all the patients since the correction of the anemia is faster when the erythrpoietic agents goes along with iron administration.

Although a number of publications discuss the efficacy and safety of the 2 erythropoietic agents, few publications compare the two. We have found only 2 retrospective studies comparing epoetin alfa and darbepoetin alfa.

The study by Reeves et al. (23,24) provides a retrospective analysis of 512 patients with chemotherapy-induced anemia who received treatment with erythropoietic agents: 196 patients received darbepoetin alfa, 212 patients received epoetin alfa, and 104 patients received both agents. Darbepoetin alfa was most frequently given in doses of 100 mcg/week and 200 mcg/2 weeks (49% and 36%, respectively, 300 patients) and 86% of the 212 patients on epoetin alfa received 40,000 IU/week. The mean baseline Hb was 10.1 g/dL for the darbepoetin alfa group and 9.6 g/dL for the epoetin alfa group. Reeves et al. analyzed the results from 350 patients (a separate analysis); 97 patients were treated with darbepoetin alfa 100 mcg/week, 70 patients were treated with darbepoetin alfa 200 mcg/2 weeks, and 183 patients were treated with epoetin alfa 40,000 IU/week. The respective increases in the Hb at 5 weeks were 0.8 g/dL, 0.8 g/dL and 0.6 g/dL and, at 8 weeks, the increases were 1.1 g/dL, 1.3 g/dL, and 0.9 g/dL. The incidence of transfusions was 4%, 11%, and 14%, respectively. The conclusion of the study was that darbepoetin alfa and epoetin alfa have a comparable efficacy in the treatment of chemotherapy-induced anemia.

The second study, published by Herrington, et al. (25), retrospectively reviewed 3123 medical histories from 65 oncology hospitals and selected 1444 patients who were treated with darbepoetin alfa and 1341 who were given epoetin alfa over 12 weeks. In 61% of the patients who received darbepoetin alfa, the dose used was 200 mcg/2 weeks, and in 72% of the patients who received epoetin alfa the dose was 40,000 IU/week. Baseline Hb was 10.3 g/dL in both groups. The dose was increased in 22% of the patients treated with darbepoetin alfa and in 23% of those treated with epoetin alfa after a mean period of 6 weeks in each group. The transfusion requirements were also similar in the 2 groups: 11% in the darbepoetin alfa group and 12% in the epoetin alfa group. After 12 weeks of treatment, the increase in Hb was 1.0 g/dL in the darbepoetin group and 1.1 g/dL in the epoetin alfa group. Harrington et al. concluded that at the doses used (darbepoetin alfa 200 mcg/2 weeks and epoetin alfa 40,000 IU/week) the efficacy is clinically comparable.

In our series, the Hb at the beginning of the treatment with erythropoietic agent is 11.85 + 1.125 for group 1 and 11.76 + 1.138 for group 2; superior to the one of the studies commented previously; nevertheless our results, with respect to the increase and maintenance of the levels of Hb, are superposable to the obtained ones by them.

Schwartzberg et al. (26) published the results of a randomized study in 312 patients with breast, lung and gynecological cancer who had chemotherapy-induced anemia; 157 patients received darbepoetin alfa 200 mcg/2 weeks and 155 patients received epoetin alfa 40,000 IU/week. Overall, no significant differences were observed in the analysis of:

- mean baseline Hb (10.4 g/dL in both groups),
- the percentage of patients who reached Hb levels ≥11 g/dL (82% vs 86%),
- the mean time in which Hb ≥ 11 g/dL was achieved (5 vs 4 weeks),
- the duration of treatment with the erythropoietic treatment (9.3 vs 10.1 weeks),
- the Hb level achieved during the study (12.1 vs 12.2 g/dL),
- the increase in Hb (1.8 vs 1.6 g/dL),
- the transfusion requirements (16% vs 17%), or
- or the adverse effects related to the treatment.

This study concluded that darbepoetin alfa (200 mcg/2 weeks) and epoetin alfa (40,000 IU/week) have comparable clinical efficacy.

At the American Society of Clinical Oncology (ASCO) 41st Annual Meeting in 2005, the final results of 2 randomized studies comparing darbepoetin alfa (200 mcg/2 weeks) and epoetin alfa (40,000 IU/week) in patients with chemotherapy induced anemia, with contradictory results, were presented. In the study conducted by Waltzman et al. (27), 180 patients receiving darbepoetin alfa and 178 patients receiving epoetin alfa had a baseline Hb of 10.1 and 10.2 g/dL, respectively. The Hb increase at the end of the study was significantly higher in the epoetin alfa group (0.8 vs 1.2 g/dL), the mean time to increase the Hb level by 1 g/dL was significantly lower in the epoetin group (48 vs 35 days), and the percentage of patients with an increase in Hb ≥2 g/dL was significantly higher in the epoetin alfa group (26% vs 44% at 9 weeks and 41% vs 57%) at the end of the study. It was concluded that the index of response is greater and the time to achieve an increase in Hb of 1 g/dL is shorter in the group treated with epoetin alfa 40,000 IU/week compared with darbepoetin alfa 200 mcg/2 weeks. In the second study, Glaspy et al. (28) randomized 1220 patients, 1209 of which received more than one dose of study drug (606 patients received darbepoetin alfa 200 mcg/2 weeks and 603 received epoetin alfa 40,000 IU/week). The mean levels of baseline Hb were 10.2 g/dL in both groups. No significant differences were found in:

- the mean Hb at the end of the study (11.8 and 11.9 g/dL),
- the percentage of patients who achieved a Hb level between 11 and 13 g/dL (76% vs 81%),
- the mean time to achieve a Hb level between 11 to 13 g/dL (6 vs 5 weeks),
- the percentage of patients who maintained a Hb level between 11 to 13 g/dL (74% vs 80%), and
- the transfusion requirements (21% vs 16%).

The authors concluded the adverse effects were also similar between the groups and that both agents present similar clinical efficacy.

In Europe, the usual administration of darbepoetin alfa is 150 mcg/week, although the optimal dose of darbepoetin alfa given weekly and its therapeutically equivalent dose with epoetin alfa (29) are not yet clearly defined.

We have not found any published study comparing the efficacy of both drugs in weekly administration. In our study, we prospectively included 125 consecutive patients receiving RT or RCT with anemia, defined as Hb < 13 g/dL in males and <12 g/dL in females. The mean Hb level at study drug initiation was 11.85 g/dL in the darbepoetin alfa group and 11.76 g/dL in the epoetin alfa group (p = ns). After 4 weeks of treatment, the Hb levels increased in both groups to 13.16 g/dL in the darbepoetin alfa group and to 13.02 g/dL in the epoetin alfa group (p = ns). Of the patients treated with darbepoetin alfa, 19.4% doubled the dose compared with 23.8% in the epoetin alfa group (p = ns). The increases in the mean levels of Hb over the baseline values one month after the final dose were 2.21 g/dL and 2.46 g/dL, respectively (p = ns). The transfusion requirements were similar in the 2 groups. These dates demonstrate the efficacy of darbepoetin alpha and epoetin alpha in increasing Hb levels and agree with Reeves et al. (24) and Herrington et al. (25), in demonstrating a similar clinical effectiveness for both erythropoietic agents. Changes regarding Hb intervention values have been published as guidelines (30) for the last few years; this study stands for march 2003 standards and does not match with actual clinical practice.

## Conclusions

The results of our study demonstrate that the 2 treatments, darbepoetin alfa 150 mcg/week and epoetin alfa 40,000 IU/week, are equally effective in correcting anemia in cancer patients receiving RT or RCT treatment.

## Figures and Tables

**Table 1:** Descriptive characteristics of the sample.

Treatment group Number of patients		Group 1 N = 62	Group 2 N = 63	P
Age (mean ± SD)		67.89 ± 11.8	66.84 ± 10.78	0.606
Sex	males	45 (72.6%)	39 (61.9%)	0.140
	females	17 (27.4%)	24 (38.1%)	
Tumor	H&N	15 (24.2%)	19 (30.2%)	ns
	Breast	5 (8.1%)	4 (6.3%)	
	Rectum	11 (17.7%)	10 (15.9%)	
	Genitourinary	15 (24.2%)	14 (23.8%)	
	Lung	4 (6.5%)	4 (6.5%)	
	Gynecologic	5 (8.1%)	5 (8.1%)	
	Esophagogastric	3 (4.8%)	3 (4.8%)	
	Other	4 (6.5%)	3 (4.8%)	
Stage	I	3 (4.8%)	10 (15.9%)	0.124
	II	24 (38.7%)	15 (23.8%)	
	III	27 (43.5%)	31 (49.2%)	
	IV	4 (6.5%)	5 (7.9%)	
	Tumor recurrence	3 (98.4%)	1 (1.6%)	
	Not stated	1 (1.6%)	1 (1.6%)	
Previous oncological treatment	Neoadjuvant CTX	8 (12.9%)	8 (12.7%)	0.592
	Surgery	26 (41.9%)	29 (46%)	0.359
	Adjuvant CTX	5 (8.1%)	8 (12.7%)	0.280
	HT	9 (14.5%)	7 (11.1%)	0.408
Current oncological treatment	RT + CTX	20 (32.3%)	24 (38.1%)	0.664
	RT only	42 (67.7%)	39 (61.9%)	
Intention to treat with RT	Radical	35 (56.5%)	32 (50.8%)	0.850
	Adjuvant	19 (30.6%)	24 (38.1%)	
	Neoadjuvant	6 (9.7%)	5 (7.9%)	
	Palliative	2 (3.2%)	2 (3.2%)	

**Table 2:** Reason for the termination of treatment with the study drug.

Reason for termination, n (%)	Group 1 (Darbepoetin alfa)	Group 2 (Epoetin alfa)
Achieved a Hb > 15 g/dL during RT or RCT	20 (65.6%)	20 (65.6%)
Hb 14 g/dL after RCT treatment	23 (38.3%)	17 (27.4%)
Completed 16 weeks of study drug	4 (6.7%)	1 (1.6%)
Study drug related or unrelated adverse event	12 (19.7%)	18 (29.0%)
Patient request	4 (6.8%)	7 (11.3%)

2 patients in each group required RBC transfusions.

**Table 3:** Mean serum iron, ferritin, and % saturation levels.

Level	Group 1 (Darbepoetin alfa)	Group 2 (Epoetin alfa)	P
Serum Iron, mcg/dL (mean ± SD)			
Baseline	75.71 ± 45.56	82.16 ± 49.23	0.456
Week 4	65.35 ± 57.87	67.73 ± 48.11	0.831
Week 12	34.09 ± 8.74	67.30 ± 64.05	0.104
1 month after final dose of study drug	99.15 ± 41.28	113.04 ± 45.43	0.186
Ferritin, ng/mL (mean ± SD)			
Baseline	255.10 ± 250.86	245.12 ± 324.88	0.851
Week 4	149.66 ± 212.08	144.41 ± 233.54	0.911
Week 12	192.18 ± 413.35	97.0 ± 199.16	0.517
1 month after final dose of study drug	246.55 ± 280.76	291.90 ± 285.28	0.510
Saturation,% (mean ± SD)			
Baseline	31.98 ± 25.05	34.37 ± 27.93	0.618
Week 4	27.44 ± 32.76	25.93 ± 23.32	0.800
Week 12	13.54 ± 5.3	24.9 ± 32.12	0.261
1 month after final dose of study drug	41.65 ± 27.87	44.64 ± 22.43	0.631
